# Spiritual Well-Being, Treatment Adherence, and Self-Management in Hemodialysis Care: A Cross-Sectional Study

**DOI:** 10.3390/healthcare14193266

**Published:** 2026-10-02

**Authors:** Handan Özdemir, Sıddıka Ersoy

**Affiliations:** 1 Department of Nursing, Faculty of Health Sciences, Burdur Mehmet Akif Ersoy University, 15030 Burdur, Turkey; 2 Department of Nursing, Faculty of Health Sciences, Suleyman Demirel University, 32000 Isparta, Turkey; siddikaersoy@sdu.edu.tr

**Keywords:** hemodialysis, renal nursing, spiritual well-being, self-management, treatment adherence

## Abstract

**Background/Objectives:** Hemodialysis requires sustained patient participation in demanding treatment and self-management routines. Spiritual well-being may be a patient-centered psychosocial resource relevant to adherence and self-management, but its independent associations with these patient-reported outcomes remain uncertain. This study examined the associations of spiritual well-being with treatment adherence and self-management among adults undergoing hemodialysis. **Methods:** This single-center cross-sectional study included 72 adults receiving maintenance hemodialysis at a public hospital in Türkiye between March and May 2025. Spiritual well-being, treatment adherence, and self-management were assessed using the Spiritual Well-Being Scale, End-Stage Renal Disease Adherence Questionnaire, and Chronic Disease Self-Management Scale, respectively. Pearson correlations and two multivariable linear regression models with HC3 robust standard errors were used. Models were adjusted for age, sex, dialysis duration, and number of comorbidities. Rank-based, influence-restricted, and nonlinear sensitivity analyses were performed. **Results:** Spiritual well-being was positively correlated with treatment adherence (r = 0.822, *p* < 0.001) and self-management (r = 0.891, *p* < 0.001). In adjusted linear models, higher spiritual well-being was associated with greater treatment adherence (B = 4.338, 95% CI 3.550–5.126; *p* < 0.001; adjusted R^2^ = 0.655) and better self-management (B = 0.494, 95% CI 0.418–0.569; *p* < 0.001; adjusted R^2^ = 0.787). Sensitivity analyses confirmed the direction and statistical significance of both associations, while nonlinear models suggested attenuation at higher spiritual well-being scores. **Conclusions:** Spiritual well-being was associated with two patient-reported outcomes relevant to long-term hemodialysis care after adjustment for the measured demographic and clinical covariates. Person-centered nephrology assessment may consider spiritual well-being alongside health literacy, psychological distress, social support, and practical treatment barriers. Longitudinal multicenter studies should determine the temporal direction and clinical implications of these relationships.

## 1. Introduction

Chronic kidney disease (CKD) is a progressive and irreversible condition that represents a major global health problem [1]. In its advanced stages, CKD may progress to end-stage renal disease (ESRD), requiring renal replacement therapy to sustain life [2]. Hemodialysis is one of the most widely used forms of renal replacement therapy, but it imposes substantial physical, psychological, and practical demands on patients’ daily lives [3]. Successful hemodialysis requires sustained adherence to dialysis attendance, prescribed medications, and dietary and fluid restrictions. Failure to adhere to these treatment requirements is associated with complications, hospitalization, and poorer clinical outcomes [4,5].

Effective management of end-stage renal disease (ESRD) depends not only on medical treatment but also on patients’ ability to actively manage their condition in everyday life. Self-management includes behaviors such as symptom monitoring, adherence to treatment recommendations, communication with health professionals, coping with psychosocial demands, and maintaining routines that support disease control [6]. Because many treatment-related decisions and behaviors occur outside the dialysis unit, patients must remain actively involved in their care. Accordingly, structured self-management support has been associated with improvements in self-care, self-efficacy, and quality of life among patients undergoing hemodialysis [7].

Spiritual well-being is a multidimensional construct involving a sense of meaning and coherence, personally valued aims, connectedness, and relationships with realities extending beyond the self [8,9]. In accordance with the scale used in the present study, it includes transcendence, harmony with nature, and reduced anomie [9]. These dimensions may be relevant to how individuals understand and respond to chronic illness, particularly when treatment substantially affects daily routines, personal roles, and future expectations. Patients living with ESRD must continually adjust to physical limitations, emotional challenges, dependency on treatment, and changes in social and family life [10].

Spiritual well-being has been associated with psychological health and adjustment among individuals living with chronic illness, including those receiving hemodialysis [11,12]. Related constructs, including spiritual health, resilience, and mental well-being, have also been associated with treatment adherence in hemodialysis populations [13,14]. Similarly, self-care and self-management in chronic kidney disease have been associated with health literacy, psychosocial resources, and structured support [15,16]. However, spiritual well-being, treatment adherence, and self-management are conceptually related but not interchangeable. Treatment adherence refers to following agreed treatment recommendations, whereas self-management additionally encompasses symptom monitoring, coping, communication with health professionals, and confidence in managing one’s condition. From a healthcare-delivery perspective, identifying psychosocial correlates of these outcomes may inform person-centered assessment, patient education, renal nursing practice, and referral within multidisciplinary hemodialysis care. Therefore, this study examined whether spiritual well-being was independently associated with treatment adherence and self-management among adults undergoing hemodialysis. We hypothesized that higher spiritual well-being would be independently associated with better treatment adherence and self-management.

## 2. Materials and Methods

### 2.1. Study Design and Setting

This cross-sectional study was conducted between 10 March and 11 May 2025 in the hemodialysis unit of a public hospital in southern Türkiye. The unit provides maintenance hemodialysis for adults with end-stage renal disease (ESRD). The study was reported in accordance with the Strengthening the Reporting of Observational Studies in Epidemiology (STROBE) Statement for cross-sectional studies [17]. A completed STROBE checklist is provided in Supplementary Table S2.

### 2.2. Participants and Sampling

During the study period, the hemodialysis unit treated 89 patients. Eligibility criteria were age ≥ 18 years, receipt of maintenance hemodialysis for at least 6 months, absence of a physical, cognitive, or mental health condition that would prevent participation, ability to communicate verbally or in writing, and provision of informed consent. Patients who did not meet any eligibility criterion, could not complete the questionnaires because of a health or communication problem, declined participation, or developed a serious clinical condition during recruitment were excluded.

**Figure 1:**
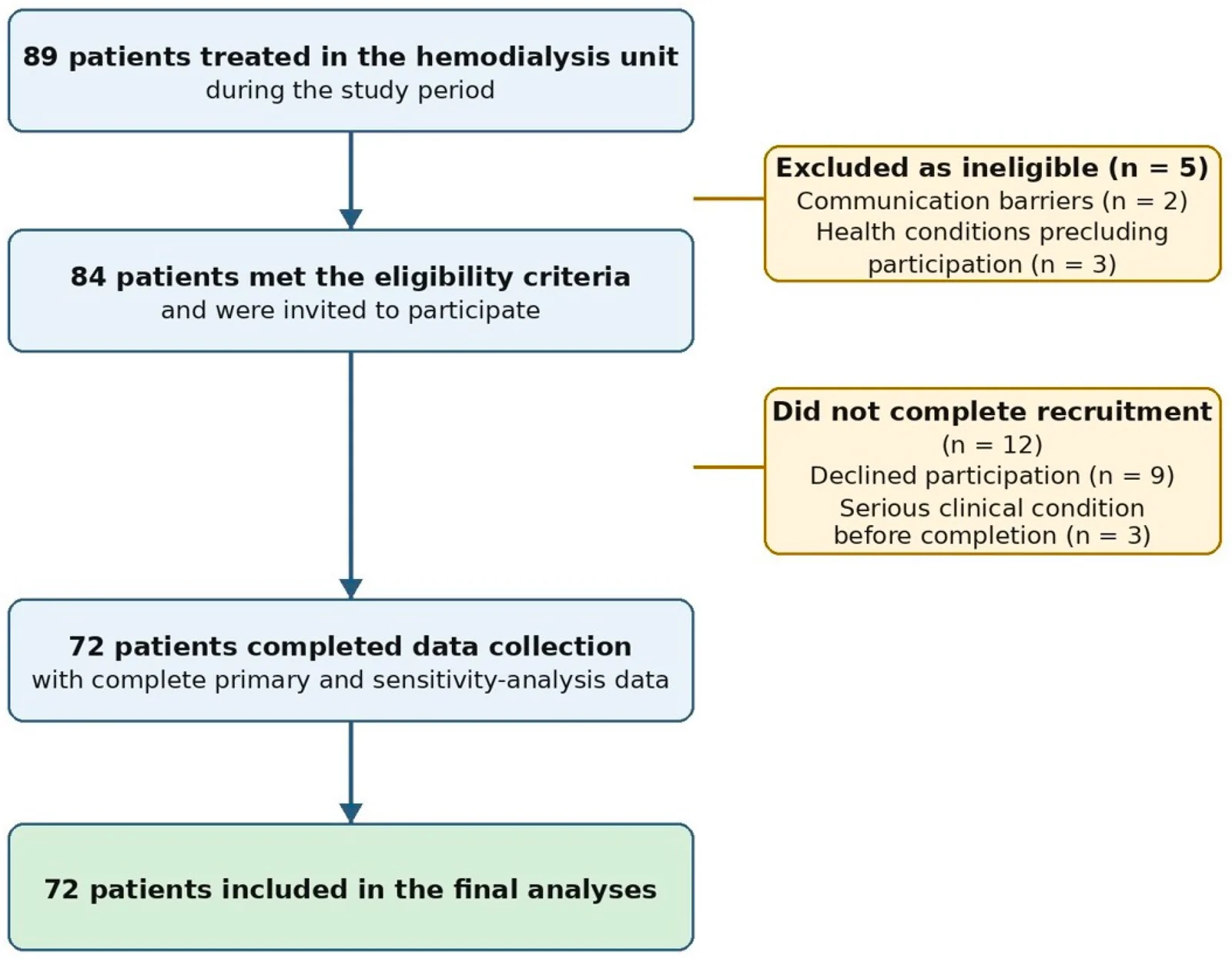
Participant flow through eligibility assessment, recruitment, data collection, and analysis.

A census approach was used: all patients treated in the unit during the data-collection period were assessed, and all who met the eligibility criteria were invited. Of 89 patients, five did not meet the eligibility criteria (two had communication barriers and three had health conditions that precluded participation), nine declined, and three developed serious clinical conditions before data collection could be completed. The final sample therefore comprised 72 patients, corresponding to 80.9% of the unit population and 85.7% of the 84 patients who initially met the eligibility criteria. All 72 participants provided complete data for the primary and sensitivity analyses (Figure 1). No formal sample size calculation was performed because the study sought to recruit the entire accessible population.

### 2.3. Data Collection

Data were collected by the research team through face-to-face interviews during routine hemodialysis sessions. S.E., who was not a member of the patients’ routine care team, administered the questionnaires. This separation was intended to reduce perceived pressure to provide favorable answers or concern that responses about treatment behavior might affect clinical care. Eligible patients received verbal and written information about the study, were informed that participation was voluntary and would not affect their treatment, and provided written informed consent before data collection. Questionnaires were completed during the first 1–1.5 h of dialysis to minimize fatigue and maintain participant comfort; each interview lasted approximately 25–30 min.

### 2.4. Patient Information Form

Sociodemographic and clinical data were collected using a Patient Information Form developed by the researchers. The form included age, sex, place of residence, living arrangement, primary caregiver, dialysis duration, presence of comorbid disease other than chronic kidney disease, and number of chronic diseases.

### 2.5. Spiritual Well-Being Scale (SWBS)

Spiritual well-being was assessed using the Spiritual Well-Being Scale developed and validated in Turkish by Ekşi and Kardaş [9]. The scale contains 29 items across transcendence, harmony with nature, and anomie. Items are rated from 1 (“not appropriate for me at all”) to 5 (“completely appropriate for me”). The prescribed reverse scoring was applied to negatively worded items, and item coding was rechecked against the validated Turkish scoring key before analysis. Total scores range from 29 to 145, with higher scores indicating greater spiritual well-being. The total score was used as the principal explanatory variable. Although the scale has Turkish validity evidence, it has not been specifically validated in a hemodialysis population.

### 2.6. End Stage Renal Disease Adherence Questionnaire (ESRD-AQ)

Treatment adherence was assessed using the End Stage Renal Disease Adherence Questionnaire (ESRD-AQ), which was developed specifically for patients receiving in-center hemodialysis [18]. The questionnaire uses heterogeneous item formats and clinically weighted scoring to assess dialysis attendance, medication use, fluid restriction, and dietary restriction. The prescribed scoring algorithm yields a total score from 0 to 1200, with higher scores indicating better adherence; this total was the treatment-adherence outcome. Because the total is a weighted composite of distinct behaviors rather than a unidimensional reflective scale, Cronbach’s alpha was not calculated or interpreted for the ESRD-AQ. The psychometric properties of the Turkish version were established among patients receiving hemodialysis [19].

### 2.7. Chronic Disease Self-Management Scale (CDSMS)

Self-management was assessed using the Chronic Disease Self-Management Scale (CDSMS), originally developed by Ngai et al. [20] and subsequently adapted and validated in Turkish by Öztürk et al. [21]. The 21-item Turkish version covers self-stigma, treatment adherence, coping with stigma, and health-care efficacy. Items are rated from 1 (“never”) to 5 (“always”). Negatively worded items were reverse-scored according to the Turkish scoring key, and all item codes and computed totals were rechecked against the raw dataset. Total scores range from 21 to 105, with higher scores indicating better overall self-management. Because the CDSMS includes a five-item treatment-adherence subscale, a sensitivity outcome was also calculated by summing the remaining 16 items (possible range, 16–80) to examine self-management after removing adherence-related content.

### 2.8. Statistical Analysis

Data were analyzed using SPSS version 25.0 (IBM Corp., Armonk, NY, USA), and regression estimates and sensitivity analyses were independently reproduced from the de-identified study dataset. Continuous variables were summarized as mean ± standard deviation (SD), and categorical variables as frequencies and percentages. Data completeness and score ranges were verified before analysis. Distributions were evaluated using histograms, Q–Q plots, skewness, and kurtosis. Pearson correlation coefficients were calculated among spiritual well-being, treatment adherence, and self-management, and internal consistency was evaluated using Cronbach’s alpha. Two separate multivariable linear regression models were fitted, with treatment adherence and self-management as the respective dependent variables. Spiritual well-being was the principal explanatory variable in both models. Age, sex, dialysis duration, and number of comorbidities were included a priori as clinically relevant covariates available in the dataset. HC3 heteroscedasticity-consistent standard errors and 95% confidence intervals were reported. Linearity was assessed using residual and component-plus-residual plots, and multicollinearity using variance inflation factors. Potentially influential observations were identified using Cook’s distance > 4/n, leverage > 2p/n, and externally studentized residuals with an absolute value > 3. Sensitivity analyses comprised Spearman rank correlations, refitting models after excluding observations exceeding the Cook’s distance threshold, and models containing a mean-centered quadratic spiritual well-being term. Unstandardized coefficients (B), robust standard errors (SE), standardized coefficients (β), *p* values, and adjusted R^2^ values were reported. The two primary outcomes were prespecified, and statistical significance was set at *p* < 0.05.

## 3. Results

The mean age of the participants was 57.36 ± 13.03 years, and the mean dialysis duration was 9.22 ± 4.17 years. Thirty-two patients were female (44.4%) and 40 were male (55.6%). Most participants lived in a city center (54.2%), followed by districts (37.5%) and villages (8.3%). All participants lived with family members and reported that their care was provided by family members. In addition to chronic kidney disease, 58 patients (80.6%) reported at least one other chronic disease. The mean number of additional chronic diseases was 1.44 ± 0.92 (Table 1). There were no missing observations for the variables included in the analyses.

The mean spiritual well-being score was 109.81 ± 25.30, the mean treatment adherence score was 938.54 ± 133.28, and the mean self-management score was 84.81 ± 14.01. After confirmation of item coding and reverse scoring, Cronbach’s alpha was 0.992 for the SWBS and 0.978 for the CDSMS. Spiritual well-being was positively correlated with treatment adherence (r = 0.822, *p* < 0.001) and self-management (r = 0.891, *p* < 0.001). Treatment adherence was also positively correlated with self-management (r = 0.818, *p* < 0.001) (Table 2).

In the adjusted linear model, spiritual well-being was associated with treatment adherence after adjustment for the measured covariates (B = 4.338, HC3 SE = 0.395, 95% CI 3.550–5.126; *p* < 0.001; β = 0.823). Age, sex, dialysis duration, and number of comorbidities were not associated with adherence. The model accounted for 67.9% of the observed variance (R^2^ = 0.679; adjusted R^2^ = 0.655). In the self-management model, spiritual well-being was also associated with the outcome after adjustment for the measured covariates (B = 0.494, HC3 SE = 0.038, 95% CI 0.418–0.569; *p* < 0.001; β = 0.892); none of the covariates was associated with self-management. This model accounted for 80.2% of the observed variance (R^2^ = 0.802; adjusted R^2^ = 0.787) (Table 3). Variance inflation factors ranged from 1.01 to 3.11. Influence diagnostics identified five observations above the Cook’s distance threshold in the adherence model and six in the self-management model; two observations exceeded the leverage threshold in each model, and one externally studentized residual exceeded an absolute value of 3 in the adherence model. The large coefficients and R^2^ values describe statistical association in this sample and should not be interpreted as clinical effect sizes or evidence of causation.

Rank-based analyses supported the primary findings: spiritual well-being correlated with treatment adherence (Spearman’s ρ = 0.786, *p* < 0.001) and self-management (ρ = 0.838, *p* < 0.001). Excluding observations above the Cook’s distance threshold modestly increased rather than attenuated the estimates for adherence (n = 67; B = 4.645, 95% CI 3.923–5.367; *p* < 0.001; adjusted R^2^ = 0.756) and self-management (n = 66; B = 0.504, 95% CI 0.440–0.568; *p* < 0.001; adjusted R^2^ = 0.835), indicating that the identified observations did not account for the associations (Supplementary Table S1).

**Figure 2:**
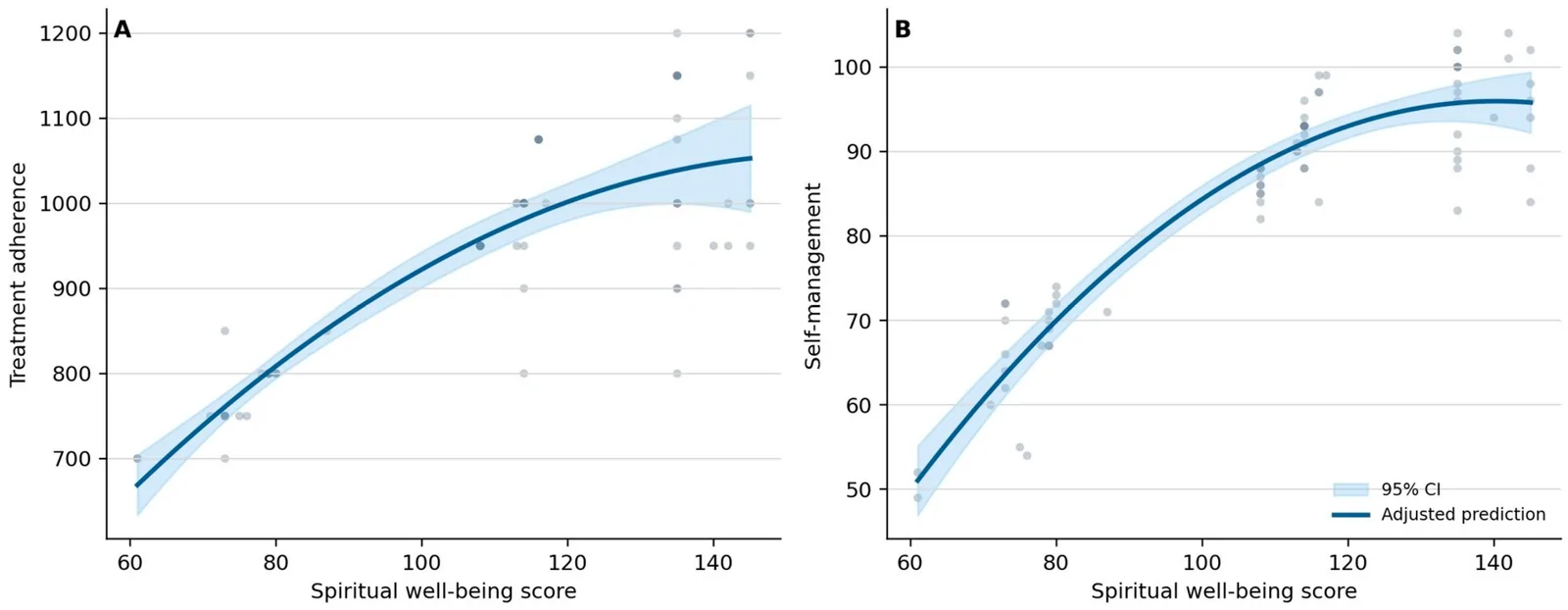
Adjusted nonlinear associations of spiritual well-being with treatment adherence (**A**) and self-management (**B**). Curves show average marginal predictions from models containing mean-centered linear and quadratic spiritual well-being terms and adjusted for age, sex, dialysis duration, and number of comorbidities; shaded bands indicate 95% confidence intervals. Points show observed values.

The quadratic models provided a better description of the upper score range. The negative squared terms were significant for treatment adherence (B = −0.0429, 95% CI −0.0744 to −0.0113; *p* = 0.008; adjusted R^2^ = 0.684) and self-management (B = −0.00714, 95% CI −0.00932 to −0.00496; *p* < 0.001; adjusted R^2^ = 0.870). Thus, both outcomes increased with spiritual well-being across most of the observed range, but the slopes flattened at higher scores (Figure 2). Linear coefficients should therefore be interpreted as average slopes rather than constant increments across the scale.

When the five-item CDSMS treatment-adherence subscale was removed, the remaining 16-item score had a mean of 66.57 ± 11.25 (observed range, 39–80; Cronbach’s α = 0.974). Spiritual well-being remained correlated with this reduced score (r = 0.846; Spearman’s ρ = 0.767; both *p* < 0.001) and remained associated after adjustment for the measured covariates (B = 0.377, HC3 SE = 0.034, 95% CI 0.309–0.445; *p* < 0.001; adjusted R^2^ = 0.705). The association therefore persisted after adherence-related CDSMS content was excluded, although the high alpha and common self-report format still require caution.

## 4. Discussion

Spiritual well-being was associated with treatment adherence and self-management after adjustment for the measured demographic and clinical covariates. The associations persisted in rank-based, influence-restricted, and adherence-excluded analyses. The nonlinear models added an important qualification: increases in both outcomes became smaller at higher spiritual well-being scores. The results therefore support graded but nonconstant associations within this sample, not uniform clinical gains for each one-point increase. Because all variables were measured concurrently, the findings do not establish whether spiritual well-being precedes treatment-related behavior, develops alongside successful disease management, or reflects other personal and social resources associated with both outcomes.

Previous hemodialysis studies have linked spiritual health or spiritual well-being with adherence, resilience, mental well-being, and psychological adaptation [11,12,13,14]. The present study therefore does not claim that the spiritual well-being–adherence relationship is itself novel. Its contribution is the concurrent evaluation of two related but distinct patient-reported outcomes, covariate-adjusted and robustness analyses, explicit testing of nonlinearity, and demonstration that the association with broader self-management persisted after removal of the CDSMS adherence subscale. Spiritual resources may help some patients preserve hope, coherence, and personally meaningful goals while coping with dialysis demands. However, hope, meaning-making, and coping were not directly measured and cannot be considered established mechanisms.

The association between spiritual well-being and self-management extends the findings beyond treatment adherence alone. Self-management encompasses the activities through which patients monitor their condition, respond to symptoms, communicate with health professionals, cope with psychosocial consequences, and integrate treatment requirements into everyday life. Among patients undergoing hemodialysis, health literacy, self-efficacy, self-regulation, and social support have been associated with self-management behavior [22]. Health literacy has also been associated with self-care behavior in the broader chronic kidney disease population [15]. Within this multidimensional context, spiritual well-being may represent an additional psychosocial correlate. However, because these potentially important cognitive and social factors were not included in the models, the independent contribution of spiritual well-being beyond them remains uncertain.

Treatment adherence and self-management are related but non-equivalent outcomes. The ESRD-AQ focuses on dialysis attendance, medication use, and dietary and fluid restrictions [18]. The CDSMS addresses broader behavioral and psychosocial domains, including coping with stigma and efficacy in interactions with health professionals, but it also includes a treatment-adherence subscale [20,21]. Shared adherence-related content may therefore have increased the correlation between the original totals. Importantly, spiritual well-being remained associated with the 16-item CDSMS score after the adherence subscale was removed. This sensitivity analysis reduces direct content overlap with the ESRD-AQ, although it does not eliminate common method variance or establish complete construct separation.

The magnitude and shape of the associations require cautious interpretation. All principal variables were self-reported in the same session and used similar response formats, creating potential common method variance [23]. Self-reported adherence may differ from behavioral or clinical indicators and is susceptible to recall and socially desirable responding [24]. Questionnaire administration by a researcher outside the routine care team may have reduced perceived pressure, but it could not remove these biases. The very high SWBS and CDSMS alpha values were confirmed after coding checks; they may reflect highly homogeneous or partly redundant content rather than measurement quality alone. The nonlinear flattening at higher scores may also indicate upper-range compression. Thus, the high correlations and explained variance may reflect both substantive relationships and shared measurement features.

Treatment adherence and self-management in hemodialysis are influenced by a complex interplay of clinical, psychological, cognitive, socioeconomic, and treatment-related factors. Common barriers include limited health literacy, psychosocial difficulties, health beliefs, medication burden, and practical challenges in integrating treatment into everyday life [16,25]. Previous studies have also highlighted the contributions of hope, social support, and individual patient characteristics to self-management and adherence behaviors [26,27]. Within this broader context, spiritual well-being should be regarded as one of several psychosocial correlates rather than the principal explanation for treatment adherence or self-management.

The findings may have implications for person-centered assessment, but they do not demonstrate that spiritual care interventions improve adherence or self-management. Assessment of spiritual well-being may be considered as part of person-centered nephrology care when spiritual concerns are important to the patient, provided that such discussions are voluntary, culturally responsive, and consistent with individual preferences. Attention to patients’ spiritual well-being may complement interventions addressing health literacy, psychological distress, social support, and practical treatment barriers. Structured self-management support has been associated with improvements in self-care, self-efficacy, and quality of life among patients undergoing hemodialysis [7]. The potential effects of interventions designed to enhance spiritual well-being on adherence, self-management, and other patient outcomes should be evaluated in prospective interventional studies before being incorporated into routine clinical practice.

Future studies should use longitudinal designs to establish temporal ordering and should complement self-reported adherence with missed or shortened dialysis sessions, interdialytic weight gain, medication records, and bio-chemical indicators [24]. Measures that separate adherence from other self-management domains would reduce content overlap, and population-specific validation of spiritual well-being measures would strengthen inference. Larger multicenter studies should prespecify models that include education, socioeconomic status, depressive symptoms, anxiety, social support, health literacy, dialysis adequacy, and detailed comorbidity burden. They should also assess patient experience, equity of access, hospitalization, and the feasibility and sustainability of voluntary, culturally responsive spiritual assessment or referral pathways.

Several limitations should be acknowledged. The cross-sectional design precluded temporal and causal inference, while the modest single-center convenience sample limited statistical precision, subgroup analyses, and generalizability. No formal sample size calculation was performed. Although the models were adjusted for four clinically relevant characteristics, residual confounding by unmeasured psychosocial, socioeconomic, and clinical factors remains possible. The concurrent use of self-report instruments may have increased susceptibility to common method variance and reporting bias. Partial content overlap between the ESRD-AQ and CDSMS further limits the interpretation of adherence and self-management as fully independent outcomes. The primary models summarized average linear associations, whereas sensitivity analyses identified attenuation at higher spiritual well-being scores. Finally, although the Spiritual Well-Being Scale has demonstrated validity in Turkish populations, it has not been specifically validated for patients undergoing hemodialysis.

## 5. Conclusions

In this modest single-center cross-sectional sample, spiritual well-being was associated with patient-reported treatment adherence and self-management after adjustment for the measured covariates. The associations persisted after influential observations were excluded and after the CDSMS adherence subscale was removed, but they attenuated at higher spiritual well-being scores. These preliminary findings do not establish causality, clinical benefit, or independence from unmeasured psychosocial and socioeconomic factors. Larger longitudinal multicenter studies using objective adherence indicators and clearly differentiated constructs are required before clinical pathways or interventions can be recommended.

## Figures and Tables

**Table 1:** Sociodemographic and clinical characteristics of patients undergoing hemodialysis (*n* = 72).

Variable	Category	*n*	%
Gender	Male	40	55.6
	Female	32	44.4
Place of residence	City centre	39	54.2
	District	27	37.5
	Village	6	8.3
Living arrangement	With family	72	100
Primary caregiver	Family member	72	100
Comorbid disease	Yes	58	80.6
	No	14	19.4
**Variable**	**Mean ± SD**		
Age, years	57.36 ± 13.03		
Dialysis duration, years	9.22 ± 4.17		
Number of chronic diseases	1.44 ± 0.92		

**Table 2:** Descriptive statistics, internal consistency, and bivariate correlations among spiritual well-being, treatment adherence, and self-management in patients undergoing hemodialysis.

Variable	Mean ± SD	Cronbach’s α	1	2	3
Spiritual well-being	109.81 ± 25.30	0.992	1		
Treatment adherence	938.54 ± 133.28	0.690	0.822 *	1	
Self-management	84.81 ± 14.01	0.978	0.891 *	0.818 *	1

* *p* < 0.001. Cronbach’s alpha was not reported for the ESRD-AQ because its weighted, multidomain total does not assume a unidimensional reflective scale.

**Table 3:** Multivariable linear regression models for treatment adherence and self-management in patients undergoing hemodialysis (*n* = 72).

Outcome	Predictor	B	HC3 SE	β	95% CI	*p*	Adjusted R^2^
Treatment adherence	Spiritual well-being	4.338	0.395	0.823	3.550 to 5.126	<0.001	0.655
	Age	0.596	1.194	0.058	−1.788 to 2.980	0.619	
	Male sex	5.290	20.987	0.020	−36.611 to 47.192	0.802	
	Dialysis duration	0.880	2.369	0.028	−3.851 to 5.610	0.712	
	Number of comorbidities	−7.323	16.037	−0.050	−39.342 to 24.698	0.649	
Self-management	Spiritual well-being	0.494	0.038	0.892	0.418 to 0.569	<0.001	0.787
	Age	−0.062	0.129	−0.057	−0.318 to 0.195	0.633	
	Male sex	−1.084	1.639	−0.039	−4.356 to 2.188	0.511	
	Dialysis duration	0.331	0.222	0.099	−0.112 to 0.774	0.140	
	Number of comorbidities	0.679	1.625	0.045	−2.566 to 3.924	0.677	

HC3 heteroscedasticity-consistent standard errors are reported. Sex was coded as male versus female. β = standardized regression coefficient. Each model included all predictors shown.

## Data Availability

The data supporting the findings of this study are available from the corresponding author upon reasonable request.

## Supplementary Materials

The following supporting information can be downloaded at https://www.mdpi.com/article/10.3390/healthcare14193266/s1, Table S1: Sensitivity analyses for the associations of spiritual well-being with treatment adherence and self-management; Table S2: Completed Strengthening the Reporting of Observational Studies in Epidemiology (STROBE) checklist for cross-sectional studies.

## Abbreviations

The following abbreviations are used in this manuscript:

CKD	Chronic kidney disease
ESRD	End-stage renal disease
STROBE	Strengthening the Reporting of Observational Studies in Epidemiology
SWBS	Spiritual Well-Being Scale
ESRD-AQ	End-Stage Renal Disease Adherence Questionnaire
CDSMS	Chronic Disease Self-Management Scale
SPSS	Statistical Package for the Social Sciences
SD	Standard deviation
HC3	Heteroscedasticity-consistent covariance matrix estimator, type 3
SE	Standard error
CI	Confidence interval
