# Supplementary material for: Spiritual Well-Being, Treatment Adherence, and Self-Management in Hemodialysis Care: A Cross-Sectional Study
Source: Healthcare (Basel). 2026 Oct 2;14(19):3266. doi: 10.3390/healthcare14193266 (PMC13665502; doi:10.3390/healthcare14193266)
Supplement: Supplementary file 1 [file healthcare-14-03266-s001.zip › healthcare-4541312-supplementary.pdf]

Supplementary Table S1. Sensitivity analyses for the associations of spiritual well-being with treatment adherence and self-management.

Outcome	Analysis/term	n	Estimate	HC3 SE	95% CI	p	Adjusted R^2
Treatment adherence	Spearman correlation	72	ρ = 0.786	—	—	<0.001	—
Self-management	Spearman correlation	72	ρ = 0.838	—	—	<0.001	—
Treatment adherence	Influence-restricted model	67	B = 4.645	0.361	3.923 to 5.367	<0.001	0.756
Self-management	Influence-restricted model	66	B = 0.504	0.032	0.440 to 0.568	<0.001	0.835
Treatment adherence	Quadratic model: linear term	72	B = 3.988	0.466	3.057 to 4.919	<0.001	0.684
Treatment adherence	Quadratic model: squared term	72	B = −0.0429	0.0158	−0.0744 to −0.0113	0.008	
Self-management	Quadratic model: linear term	72	B = 0.435	0.031	0.373 to 0.498	<0.001	0.870
Self-management	Quadratic model: squared term	72	B = −0.00714	0.00109	−0.00932 to −0.00496	<0.001	

Spiritual well-being was mean-centered before calculation of the squared term. Influence-restricted models excluded observations with Cook's distance >4/n. All regression models retained age, sex, dialysis duration, and number of comorbidities and used HC3 heteroscedasticity-consistent standard errors.

Supplementary Table S2. Completed Strengthening the Reporting of Observational Studies in Epidemiology (STROBE) checklist for cross-sectional studies.

Section	Reporting recommendation	Location in manuscript
Title and abstract	Identify the study design in the title or abstract.	Title; Abstract
	Give a balanced summary of the methods and findings.	Abstract
Introduction	Explain the scientific background and rationale.	Section 1, paragraphs 1-4
	State the objectives and any prespecified hypotheses.	Section 1, final paragraph
Methods	Present the key elements of the design early.	Section 2.1
	Describe setting, location, and relevant dates.	Section 2.1
	Give eligibility criteria and participant sources and selection methods.	Section 2.2; Figure 1
	Define outcomes, exposure/predictor variables, confounders, and effect modifiers.	Sections 2.4-2.8
	Describe the data sources and measurement methods for each variable.	Sections 2.3-2.7
	Describe efforts to address potential sources of bias.	Sections 2.2-2.3 and 2.8; Section 4
	Explain how the study size was determined.	Section 2.2
	Explain how quantitative variables were handled in analysis.	Section 2.8
	Describe statistical methods, including control of confounding.	Section 2.8
	Describe methods used to examine subgroups and interactions.	Not applicable; no subgroup or interaction analyses
	Explain how missing data were addressed.	Sections 2.2 and 3
	If applicable, describe analytical methods accounting for the sampling strategy.	Not applicable; census approach in one unit
	Describe sensitivity analyses.	Section 2.8; Supplementary Table S1
Results	Report participant numbers at each study stage.	Section 2.2; Figure 1
	Give reasons for non-participation at each stage.	Section 2.2; Figure 1
	Use a flow diagram where helpful.	Figure 1
	Report participant characteristics and relevant covariates.	Section 3; Table 1
	State the number of participants with missing data for variables of interest.	Sections 2.2 and 3
	Report outcome data or summary measures.	Section 3; Table 2
	Give unadjusted and adjusted estimates with precision and identify adjusted covariates.	Section 3; Table 3
	Report category boundaries when continuous variables are categorized.	Not applicable
	When relevant, translate relative risk estimates into absolute risk for a meaningful period.	Not applicable
	Report other analyses, including sensitivity analyses.	Section 3; Supplementary Table S1
Discussion	Summarize the main results in relation to the objectives.	Section 4, first paragraph
	Discuss study limitations, including direction and magnitude of possible bias.	Section 4, limitations paragraph
	Provide a cautious overall interpretation considering objectives, limitations, and other evidence.	Sections 4 and 5
	Discuss the generalizability of the findings.	Section 4, limitations and future-study paragraphs
Other information	Report funding sources and the role of funders.	Funding statement
